# Acute respiratory infection and bacteraemia as causes of non-malarial febrile illness in African children: a narrative review

**DOI:** 10.15172/pneu.2015.6/488

**Published:** 2015-12-01

**Authors:** Florid Muro, Rita Reyburn, Hugh Reyburn

**Affiliations:** 13 Kilimanjaro Christian Medical Centre, Moshi, Tanzania; 23 Murdoch Children’s Research Institute, Royal Children’s Hospital, Parkville, Victoria Australia; 33 Department of Disease Control, London School of Hygiene and Tropical Medicine, Keppel St London, WICE7HT UK; 43 New Vaccine Evaluation Project, Colonial War Memorial Hospital, Suva, Fiji

**Keywords:** Respiratory, inflection, non-malaria, Africa, child

## Abstract

The replacement of “presumptive treatment for malaria” by “test before treat” strategies for the management of febrile illness is raising awareness of the importance of knowing more about the causes of illness in children who are suspected to have malaria but return a negative parasitological test. The most common cause of non-malarial febrile illness (NMFI) in African children is respiratory tract inflection. Whilst the bacterial causes of NMFI are well known, the increasing use of sensitive techniques such as polymerase chain reaction (PCR) tests is revealing large numbers of viruses that are potential respiratory pathogens. However, many of these organisms are commonly present in the respiratory tract of healthy children so causality and risk factors for pneumonia remain poorly understood. Inflection with a combination of viral and bacterial pathogens is increasingly recognised as important in the pathogenesis of pneumonia. Similarly, blood stream inflections with organisms typically grown by aerobic culture are well known but a growing number of organisms that can be identified only by PCR, viral culture, or serology are now recognised to be common pathogens in African children. The high mortality of hospitalised children on the first or second day of admission suggests that, unless results are rapidly available, diagnostic tests to identify specific causes of illness will still be of limited use in guiding the potentially life saving decisions relating to initial treatment of children admitted to district hospitals in Africa with severe febrile illness and a negative test for malaria. Malaria control and the introduction of vaccines against *Haemophilus influenzae* type b and pneumococcal disease are contributing to improved child survival in Africa. However, increased parasitological testing for malaria is associated with increased use of antbiotics to which resistance is already high.

## 1. Introduction

Interest in non-malarial febrile illness (NMFI) has increased following more widespread use of parasitological testing for malaria and, in particular, since 2010 when the World Health Organization (WHO) recommendation for antimalarial treatment for any unexplained fever was replaced by the recommendation that, wherever possible, antimalarial treatment should be restricted to children with a positive parasitological test [1,2]. Support for this policy by a number of major funders, including the Global Fund to Fight AIDS, Tuberculosis and Malaria, is having a major impact on case management of fevers in Africa. In the WHO Africa region, the number of rapid diagnostic tests (RDTs) for malaria that were distributed through national malaria control programmes rose from less than 2 million in 2006 to more than 80 million in 2012 [3]. Over the same period, the proportion of suspected malaria cases that received a diagnostic test rose from an estimated 20% to 60% [3]. Consequently, these developments are generating large numbers of patients who were previously treated for malaria but are now being considered for treatment of an alternative cause of fever [4].

The definition of NMFI is pragmatic but it remains a useful term in a period of transition from presumptive to more targeted treatment of febrile illness in African children. The term describes the situation of a patient with suspected malaria and a negative parasitological test. Like many causes of fever, malaria is an illness with clinical features that often overlap with other common causes of fever [5]. The most common of these is respiratory tract infection since fever can cause rapid breathing in children [6–8], and acidosis (commonly found in children with severe malaria) can cause deep laboured breathing with abnormal chest wall movement [8,9]. In addition, the features of cerebral malaria can be similar to those of meningitis [10], and fever without specific features can resemble non-severe malaria [11]. Diarrhoeal diseases rarely cause diagnostic confusion and so are not considered under NMFI. Similarly, otitis media or urinary tract infections usually present with clinical features that generally do not overlap with those of malaria. Low density *Plasmodium* parasitaemia may occur in the neonatal period but is usually asymptomatic due to the presence of maternal antibody and rarely meets our definition of NMFI [12]. Thus neonatal pneumonia, an important but specialised topic, is outside the scope of this review.

This review describes the main causes of NMFI in African children, with an emphasis on acute lower respiratory tract infections. Systematic testing of children for human immunodeficiency virus (HIV) is still not commonly practiced in Africa so there are few studies comparing causes of fever in children with or without HIV. The prevalence of HIV in hospitalised children varies between 1% and 17% in areas highly endemic for malaria but is considerably less in children presenting to outpatient clinics [13,14]. In this review, HIV-infected children have not been excluded and HIV data are shown where available.

## 2. Diagnostic accuracy, overlapping definitions, and co-infection

A definitive diagnosis of malaria is often difficult due to a number of reasons [15], the best known of which is that *Plasmodium* parasitaemia may be present as an incidental finding in a child with fever due to another cause. Determination of parasite density, which influences the attributable fraction of fever due to malaria, can be of help: The presence of parasites at high density make malaria more likely to be the cause of a child’s illness than a low density infection, but this relationship is influenced by the level of malaria endemicity [16,17]. In addition, blood slide diagnosis may miss true *P. falciparum* infections due to either parasite sequestration or very low density parasitaemia, although true “slide-negative malaria” is probably rare. RDTs for malaria are not affected by parasite sequestration since they measure water-soluble parasite antigens but, in areas of high transmission, devices that detect histidine-rich protein-2 (HRP-2) may lack specificity due to the persistence of HRP-2 in blood for up to 6 weeks following infection [18,19]. Finally, up to 10% of children with severe malaria also have a blood stream bacterial infection, a fact that has led some to recommend antibacterial treatment of all children with severe malaria [13,20]. While some of these cases may be due to incidental parasitaemia, it is now well established that malaria itself is an important risk factor for non-typhi *Salmonella* (NTS) and other Gram-negative infections [21,22].

## 3. Pneumonia in African children

Pneumonia is the most common alternative diagnosis to malaria in a febrile child in Africa [8,9]. The global incidence of hospital admissions of children with severe ALRI (including pneumonia and bronchiolitis) is age-dependent: it is highest in children under the age of 2 years [23] and substantially higher in developing compared to industrialised countries, but similar between Asia and Africa [23,24]; however, hypoxaemia in children meeting WHO definitions of pneumonia severity [25] is more common in Asia [26] (possibly due to misclassification of children with malarial acidosis as severe pneumonia). Thirty-eight percent of childhood cases of severe ALRI do not reach hospital, and 81% of those severe ALRI cases result in death: substantially higher than in children hospitalised with severe ALRI [23]. This estimate was based on verbal autopsy and should therefore be treated with caution.

### 3.1 Pneumonia case definitions

The study of pneumonia suffers from, among other things, difficulties in obtaining a culture specimen from the site of infection and variation in case definitions [27], the former of which is currently being, at least to some extent, addressed by the study of Pneumonia Etiology Research for Child Health (PERCH)(Table 1) [33].

**Table 1:** Studies of pneumonia in children contributing to World Health Organization (WHO) clinical case definitions

Study	Site	Sample size	Gold standard pneumonia definition	Clinical signs/symptoms investigated	Conclusions regarding definition
Shann et al [28]	Goroka, Papua New Guinea	350	Crepitations on auscultaion	Age, RR, lower chest wall indrawing, cyanosis, wheeze, pulse rate, palpable liver, temperature >37.5°C, feeds poorly	RR >50/minute was the most accurate way to differentiate pneumonia from nonpneumonia
Cherian et al [29]	Vellore, Tamil Nadu, India	682	Crepitations, wheeze, bronchial breathing or radiological abnormalities	RR, parental report of rapid breathing, intercostal retraction	Refined the value of RR by age stratification to >50 for infants and >40 for children >12 months
Campbell et al [30]	Banjul, The Gambia	222 (episodes of illness in a cohort study)	Radiological signs (lobar consolidation)	Vomiting, rapid breathing, refusing to feed, chest indrawing, RR, nasal flaring, temperature, heart rate, crepitations, bronchial breathing or reduced air entry, rhonchi, grunting	Temperature >38.5°C, refusing to feed and vomiting were the most useful predictors of severe pneumonia in infants, whereas temperature >38.5°C and RR >60/minute were the most useful among children aged 1–4 years old
Mulholland et al [31]	Philippines, Swaziland	730	Complete history, physical examination by paediatrician, and CXR	Cough, difficulty breathing, chest wall indrawing, RR. Cases with wheeze excluded	Sensitivity and specificity for RR >40/minute or for lower chest wall indrawing were between 0.77 and 0.81 in 2 different settings, but specificity was lower when judged by a healthcare worker
Simoes and McGrath [32]	Mbabane, Swaziland	362	Paediatrician’s assessment on WHO criteria	Cough, difficulty breathing, ability to drink/feed well, convulsions, abnormal sleepiness, stridor, severe undernutrition, fever, wheeze, lower chest wall indrawing, tachypnoea, fever	Using RR and lower chest wall indrawing, nurses and nursing assistants detected 71%–83% of pneumonia cases with specificity of 84%–85%

CXR, chest radiograph; RR, respiratory rate

Reproduced from Scott et al [33]

The selection of case definition depends on available diagnostic resources and priority for either high specificity (needed for clinical trials) or high sensitivity (needed for treatment of sick children) (Table 2). In most areas of Africa, the WHO definitions for severe pneumonia (cough and difficulty breathing with lower chest wall indrawing) and very severe pneumonia (cough and difficulty breathing with danger signs) [25] are generally followed, of which the definition of severe pneumonia is used most commonly. Raised respiratory rate (RR) or chest wall indrawing have a sensitivity and specificity of approximately 81% and 77%, respectively, when compared to paediatrician diagnosis of pneumonia supported by chest roentgenogram [31]. In contrast to definitions of severe pneumonia, there is only one definition of the WHO category of “non-severe pneumonia” (cough or difficulty in breathing with raised RR for age) but the significance of this as a risk factor for progression to severe pneumonia is not clear. While some studies have shown a correlation between raised RR and severe pneumonia [31,37], a placebo-controlled trial of amoxicillin in children with nonsevere pneumonia in Pakistan [38] failed to show a significant difference between trial arms, suggesting either that non-severe pneumonia may in the large majority of cases be due to a viral infection alone or the low specificity of the WHO definition of non-severe pneumonia includes many illnesses with a non-respiratory cause.

**Table 2:** Examples of varying definitions of severe pneumonia depending on purpose

Source	Very severe pneumonia	Severe pneumonia	Purpose
WHO [25]	CDB+ multiple convulsions or coma or lethargy or vomiting everything or inability to drink or cyanosis or severe respiratory distress	CDB+ chest indrawing	Treatment of children with suspected pneumonia
Cutts et al [34]		Clinically suspected pneumonia with radiological opaque or fluffy opacities in part or all of a lobe of the lung or pleural effusion	Clinical trial of pneumococcal vaccine
Nokes et al [35]	As severe but >2 criteria needed	CDB+ >1 of: intercostal indrawing, inability to feed, increased RR for age, SPO_2_ <90%	Epidemiological description of admitted cases
Scott et al [33]	CDB+ any of: hypoxaemia (SPO_2_ <90%), inability to feed, head nodding, or impaired consciousness	CDB+ chest indrawing	Epidemiological description of admitted cases
Nair et al [23]	As severe, + any IMCI danger sign or hypoxaemia (SPO_2_ <90%)	CDB+ admitted to hospital	Estimation of global burden
Mulholland et al [36]	Invasive Hib disease verified by positive isolate from blood or CSF culture		Randomised clinical trial of Hib vaccine

CDB+, cough/difficulty breathing plus; IMCI, integrated management of childhood illnesses; SpO_2_, saturation of peripheral oxygen; RR, respiratory rate; Hib, *Haemophilus influenzae* type b; CSF, cerebral spinal fluid; WHO, World Health Organization

A number of studies, summarised by Rudan et al [24], have described predictors of mortality among African children with pneumonia that include malnutrition, HIV infection, subcostal recession, and altered consciousness. The latter 2 factors are reflected in the increased mortality associated with the WHO classification of “very severe” compared to “severe” pneumonia, respectively [39]. In addition, access to care is an important determinant of survival in children with pneumonia [23].

### 3.2 Burden and causes of pneumonia in African children

Estimating incidence and causes of pneumonia in Africa is difficult due to the problems already mentioned, compounded by the few high quality laboratories in Africa and the changing epidemiology created by vaccines and availability of polymerase chain reaction (PCR) methodologies. For more than a decade, the Child Health Epidemiology Reference Group (CHERG)—a group of independent technical experts on global child morbidity and mortality estimates co-sponsored by WHO and UNICEF—has grappled with these problems. The 2013 CHERG estimates are based on a model using estimates of prevalence of known risk factors, incidence data from control arms of pneumonia clinical trials [40], and results from 28 pneumonia incidence studies that met minimum inclusion criteria [41]. Even among these estimates, the incidence of pneumonia varied 100-fold, reflecting both true variation but also lack of standardisation between studies. The CHERG estimates are summarised in Table 3. The 3 subsequent tables have been selected from published studies to illustrate specific issues with assigning causality to pneumonia in Africa, that is, the availability of PCR and the frequency of carriage of viral pathogens among healthy controls.

**Table 3:** Child Health Epidemiology Reference Group (CHERG) estimates of pneumonia in children 0–4 years of age in countries in the World Health Organization (WHO) Africa region shown as national level totals (incidence) and by causative pathogens; estimates of the number of new severe episodes (according to WHO’s definition) in the year 2010 that require hospitalisations, shown as national level totals (severe episodes, all ALRI) and by causative pathogens; and estimates of the number of child deaths attributable to pneumonia in 2011 (mortality, all ALRI) and the proportion of deaths caused by causative pathogens.

Population 0–4 years = 134,240,762			
	Number of cases	Incidence/year	% of cases
All ALRI	36,412,108	0.271 (0.213–0.338)^a^	100
Of which:			
*Streptococcus pneumoniae*	2,575,393	0.019 (0.015–0.022)	7.1
*Haemophilus influenzae* type b	780,756	0.006 (0.003–0.006)	2.1
Respiratory syncytial virus	10,501,165	0.078 (0.061–0.090)	28.8
Influenza virus	6,215,546	0.046 (0.056–0.053)	17.1
Other cause estimates not available			
Severe ALRI (severe morbidity)	4,166,781	0.031 (0.018–0.036)	100
Of which:			
*Streptococcus pneumoniae*	761,241	0.006 (0.004–0.007)	18
*Haemophilus influenzae* type b	130,134	0.001 (0.0005–0.001)	3
Respiratory syncytial virus	558,669	0.004 (0.003–0.005)	13
Influenza virus	171,931	0.001 (0.001–0.002)	4
Other cause estimates not available			
ALRI Deaths (morbidity)	531,451	0.004 (0.002–0.005)	100
Of which:			
*Streptococcus pneumoniae*	173,896	0.0013 (0.001–0.002)	33
*Haemophilus influenzae* type b	42,404	0.0003 (0.0001–0.0004)	8
Other cause estimates not available			

ALRI, acute lower respiratory infection; ^a^Interquartile range of incidences in 46 WHO-Afro countries

Adapted from Rudan et al [24]

Table 4 is reproduced from a recent study in Malawi [42] demonstrating the range of pathogens commonly identified in African children admitted to hospital with severe pneumonia, albeit in an area of relatively high HIV prevalence. In addition, this study highlights the greatly increased sensitivity of PCR in detecting respiratory pathogens, although since the upper respiratory tract is not a “normally sterile site” the interpretation of the presence of these pathogens is challenging.

**Table 4:** Aetiology of radiologically confirmed pneumonia in Malawian children in 2011 using different laboratory methods

	No.	HIV+ (%)	Blood culture	Lung aspirate culture	Lung aspirate latex	Lung aspirate PCR
Bacterial aeteology						
Total	45		8	2	4	36
*Streptococcus pneumoniae*	37	68	6	1	3	31
*Streptococcus pneumoniae* / *Streptococcus typhimurium*	2	0	2	1	0	NT
*Haemophilus infuenzae* type b	6	67	0	0	1	5
Viral aeteology						
Total	24		NT	NT	NT	24
Adenovirus	15	53	NT	NT	NT	15
Bocavirus	4	50	NT	NT	NT	4
Cytomegalovirus	3	100	NT	NT	NT	3
Atypical						
*Chlamydia pneumoniae*	2	0	NT	NT	NT	2
*Mycoplasma pneumoniae*	0		NT	NT	NT	0
*Pneumocystis jirovecii*	3	100	NT	NT	NT	3

NT, not tested; PCP, pneumocystis pneumonia; Hib, *Haemophilus infuenzae* type b; CMV, cytomegalovirus; HIV, human immunodeficiency virus; PCR, polymerase chain reaction

**Bacteria:** 2 patients had mixed infection with *S. typhimurium* and *S. pneumoniae*; 1 had *S. typhimurium* from blood culture and *S. pneumoniae* from blood PCR, and adenovirus from lung aspirate PCR, 1 had *S. typhimurium* from blood and lung aspirate culture and *S. pneumoniae* from lung aspirate PCR, 7 had *S. pneumoniae/adenovirus*, 1 had *S. pneumoniae/chlamydia*, 1 case with *S. pneumoniae* had *Mycobacterium tuberculosis* cultured from nasopharyngeal aspirate after induced sputum.

**Pneumocystis:** 1 patient had PCP/Hib, 1 patient had PCP/CMV, 1 patient had PCP/adenovirus

**Viruses:** 1 adenovirus/CMV, 1 adenovirus/chlamydia, 1 bocavirus/CMV

Reproduced from Carrol et al [42]

#### 3.2.1 Bacterial pneumonia

The incidence of pneumonia due to *Streptococcus pneumoniae* or *Haemophilus infuenzae* type b (Hib) is changing as vaccines against these infections are now being introduced across the continent. Currently, all African countries have introduced Hib conjugate vaccine in infants and all but 2 sub-Saharan African countries have introduced or are in the process of introducing infant vaccination using a 10- or 13-valent pneumococcal conjugate vaccine (PCV-10 or PCV-13) [43].

The high efficacy of these vaccines in African children has been established in clinical trials [34,36,44]. The effectiveness of the Hib conjugate vaccine is encouraging, with reports of reductions to near-zero incidence of invasive Hib disease following vaccination, although questions remain regarding the duration of protection following immunisation in infancy [45,46].

The impact of PCV-10 is still being assessed and no large studies have so far been published. In a district hospital on the Kenyan coast, paediatric admissions for invasive pneumococcal disease (IPD) from the nearby demographic surveillance system have been continuously monitored since 2003. There was a 50% reduction in admissions with IPD between 2003 and 2007 that may have been due to the reduction in malaria incidence that occurred between these years [22,47]. PCV-10 was first introduced to the childhood immunisation schedule in 2011. It is likely that PCV will bring additional benefits of secondary protection of older (unvaccinated) family members from IPD although there is also evidence of increased colonisation by non-vaccine strains of *S. pneumoniae* [46].

*Staphylococcus aureus* and Gram-negative infections are important, albeit in the minority, causes of bacterial pneumonia. Staphylococcal pneumonia is often very severe and may affect very young children. Nosocomial transmission of methicillin-resistant *S. aureus* is a particular problem in intensive care units and has been described in Ghana, although its routine detection in Africa is almost certainly hampered by lack of laboratory capacity [48]. Strains of *S. aureus* carrying the Panton-Valentine leukocidin genes cause a particularly severe form of pneumonia; while most reports of this organism are from developed countries, there is at least one case report of a fatal infection in a traveller returning from Senegal [49].

Gram-negative infections, and in particular NTS, are associated with malaria (as described below) and many of these qualify for the WHO definition of severe pneumonia. While this definition lacks specificity, there is clear evidence that these infections are responsible for true pneumonia defined by isolation of the organism from consolidated lung [50].

#### 3.2.2 Viral pneumonia

The study of viral pneumonia has been greatly enhanced by the use of PCR. A large case control study of paediatric hospital admissions for severe or very severe pneumonia (by WHO criteria) in Kenya in 2007 identified a number of viruses from a nasal wash specimen [51]. Consistent with other studies, the incidence of clinically diagnosed pneumonia was age-dependent, varying from 4.9% in the first year of life to <0.1% in children over the age of 5 years (Table 5). Respiratory syncytial virus (RSV) was detected in almost half of the infants and a third of the children with severe or very severe pneumonia, followed in frequency by human coronavirus (6.7%) and influenza virus type A (5.8%) while all others occurred at a frequency of less than 5%. In addition, RSV was responsible for a strong seasonal trend in pneumonia incidence (Table 6). Among the viruses identified, only RSV was associated with severe disease (5% in control participants; adjusted odds ratio, 6.11 [95% CI 1.65–22.6])

**Table 5:** Incidence of admission to a Kenyan district hospital with “severe pneumonia” or “very severe pneumonia” and viruses identified by polymerase chain reaction of nasal wash

Age Group	under 28 days	All <1 y	1 to 1.99 y	2 to 4.99 y	All <5 y	5 to 12.99 y	All <13 y
Denominator	9,423 births	8,837			44,538		104,505
	per 1,000 live births	per 100,000 children/y					
‘Severe’ or ‘very severe’ pneumonia	6.65	4,798	1,674	543	1,522	99	681
Any respiratory virus	3.79	2,993	871	213	862	36	380
Respiratory syncytial virus	2.46	2,038	455	85	535	15	233
Human coronavirus 229E	0.51	318	135	32	105	3	46
Influenza A	0.31	244	97	32	82	15	39
Human parainfluenza virus	0.10	212	48	11	57	6	26
Adenovirus	0.10	149	77	21	55	9	26
Human metapneumovirus	0.20	138	58	11	44	6	21

Reproduced from Berkley et al [51]

**Table 6:** Clinical features of 759 children admitted with respiratory syncytial virus and other respiratory viruses to a Kenyan district hospital in 2007

Median (IQR)			Median (IQR) *p* value^a^											
No virus N=334			Any virus N=425			RSV only N=206			RSV + another virus N=54			Non-RSV virus N=165		
Age (months)	11.3	(3.8 to 24)	7.5	(2.7 to 18)	<0.001	6.1	(2.5 to 13)	<0.001	7.5	(2.7 to 16)	0.011	10	(4.5 to 20)	0.39
Inpatient stay (days)	4	(2 to 6)	3	(2 to 5)	0.17	3	(2 to 5)	0.07	4	(3 to 6)	0.80	4	(2 to 6)	0.56
	No.	(%)	No.	(%)	*p* value^a^	No.	(%)	*p* value^a^	No.	(%)	*p* value^a^	No.	(%)	*p* value^a^
	No virus N=334		Any virus N=425			RSV only N=206			RSV + another virus N=54			Non-RSV virus		N=165
Very severe pneumonia	73	(22)	53	(13)	0.001	22	(11)	0.001	8	(15)	0.24	23	(14)	0.35
Wheeze	50	(15)	59	(14)	0.67	30	(15)	0.90	8	(15)	0.98	2	(13)	0.50
Hypoxia	40	(13)	40	(9)	0.13	18	(8.7)	0.14	8	(15)	0.70	14	(8.5)	0.14
Capillary refill ≥3 seconds	16	(4.8)	10	(2.4)	0.07	1	(0.5)	0.004	1	(1.9)	0.49	8	(4.9)	0.98
Severe anaemia	15	(4.6)	12	(2.9)	0.23	3	(1.5)	0.082	1	(1.9)	0.71	8	(4.9)	0.85
History of prematurity	17	(7.8)	14	(4.1)	0.07	5	(2.9)	0.046	2	(4.4)	0.54	7	(5.7)	0.49
Congenital heart disease	12	(3.6)	5	(1.2)	0.05	2	(1.0)	0.09	0	(0)	0.23	3	(1.8)	0.40
HIV	26	(8.0)	26	(6.2)	0.35	8	(4.0)	0.07	4	(7.6)	1	14	(8.6)	0.82
Severe malnutrition	26	(7.8)	18	(4.2)	0.04	5	(2.4)	0.009	2	(3.7)	0.40	11	(6.7)	0.65
Bacteraemia	20	(6.0)	16	(3.8)	0.15	5	(2.4)	0.056	2	(3.7)	0.75	9	(5.5)	0.81
Death	16	(4.8)	8	(1.9)	0.02	2	(1.0)	0.023	0	(0)	0.14	6	(3.6)	0.56

IQR, interquartile range; RSV, respiratory syncytial virus; HIV, human immunodeficiency virus

^a^*p* values in comparison with children with no virus

Reproduced from Berkley et al [51]

A number of case-control studies have documented that both viral and bacterial respiratory pathogens are frequently isolated from the upper respiratory tract of healthy children and this underlines the importance of including well-matched controls in any study of the causes of respiratory illness in children. RSV and influenza virus are most consistently found to be significantly associated with respiratory illness compared to controls, although the odds of association may not be strong enough to be diagnostically useful [51–54]. With regard to other viruses, it seems likely that factors such as co-infection and density of infection may need to be considered and the lack of association between cases and controls does not exclude a virus as a cause of pneumonia.

There is evidence from various sources of the association between viral and bacterial pneumonia. There are anecdotal reports of lobar pneumonia in association with the 1918 influenza pandemic and the H1N1 epidemic [55]. In a randomised, placebo-controlled trial of a 9-valent pneumococcal conjugate vaccine in South African infants, there was a 31% reduction in pneumonia associated with any of 7 respiratory viruses suggesting that infection with these viruses predisposes to pneumococcal pneumonia [56]. Immunological studies in mice suggest that infection with respiratory viruses impairs alveolar macrophage function for up to 10 days following infection and that this period was associated with severe and fatal bacterial pneumonia [57].

#### 3.2.3 Blood stream infections

A study to identify bacterial causes of illness in sub-Saharan Africa identified 22 published studies since 1980 of children admitted to hospital with a blood culture result [58]. Overall, 5,364 (12.3%) of 43,130 children had bloodstream infections. Of these, 1,643 (29.1%) non-malarial bloodstream infections were due to *Salmonella enterica* (of which 58.4% were due to NTS) and 1,031 (18.3%) were due to *S. pneumoniae*. The commonly reported organisms isolated from febrile children in Africa include *S. pneumoniae* (18.3%), NTS (17%), *S. aureus* (9.5%), and *Escherichia coli* (7.3%).

The available evidence suggests that bacteraemia is uncommon in children with non-severe illness since the prevalence of a positive aerobic blood culture is approximately 1% compared to an incidence of up to 15% among children needing hospital admission [4,13,20,59].

As alluded to above, a number of studies have described *P. falciparum* infection as a risk factor for blood stream infection with NTS and other Gram-negative infections. This association was first investigated in The Gambia where it was observed that children with malaria whose fever did not settle on quinine treatment often grew NTS from their blood culture and these infections were limited to the malaria transmission season while carriage of NTS by domestic animals was perennial [60]. Studies from Kenya and Tanzania have since found a high risk of NTS infection in children with a positive HRP-2 rapid test for malaria but a negative blood film for *Plasmodium* parasites, suggesting that vulnerability to these infections may persist for several weeks after an episode of malaria [13,20]. The association and direction of causality of malaria has been strengthened further by the finding that the protection conferred by sickle cell trait (HbAS) against NTS is mediated by the protection against malaria associated with HbAS [22,61]. Immunological studies have extended the understanding of possible mechanisms of how malaria reduces immune responses to NTS [62]. A recent comparison between areas of high and low malaria transmission has described a striking contrast in risk factors for NTS and *S. typhi*, the latter appearing to infect older, non-HIV-infected children [63,64].

#### 3.2.4 Atypical infections

In the context of this review, “atypical infection” is defined as infections not commonly isolated from aerobic blood culture. Knowledge of these infections in African children is limited, although increasing with the use of molecular diagnostics over the last decade. To investigate the frequency with which reports of these infections have been made, we used methods broadly the same as used by Reddy et al [58] and searched PubMed for reports of infection in Africa published between 1980 and 2014 of illness due to organisms not commonly isolated from blood culture. For practical reasons no age restriction was placed on the search, although papers that specifically referred to HIV-infected patients were excluded. A total of 389 reports met the inclusion criteria. The number of these reports for each infection provides only an approximate indication of incidence since the decision to report an infection depends on factors such as rarity, scientific interest, severity etc. However, evidence from a recent study in Tanzania suggests that some of these infections (e.g. leptospirosis, Q fever) may be relatively common, hence further studies including comparison with healthy controls and regional differences are indicated [65].

## 4. Discussion

The currently increasing attention to diseases that until now have been relatively neglected is encouraging, especially since a number of studies have shown that among hospitalised children with fever a negative malaria slide is generally associated with higher mortality. However, the recent attention to NMFI might lead some to think that a new epidemic has been discovered while in truth this is an “epidemic of awareness” of infections that have been present but neglected for a long time. In reality, the decline in malaria incidence has been associated with a reduction in invasive bacterial disease, particularly NTS infection. Falling incidence of malaria is by no means universal [66,67]. In many areas it is providing an opportunity to improve diagnostic accuracy and improved quality of care, but this process needs to be accompanied by improved surveillance of febrile illness and the antimicrobial susceptibilities of its causes.

### 4.1 The usefulness of knowing the causes of NMFI

There are a number of important reasons to know the local diagnostic probabilities of common causes of fever, the main one being the need for a guide to pragmatic treatment. However, there are very few effective interventions available for the treatment of sick children in African hospitals; the key treatment decision based on the likely pathogen is still limited to the use of an antibiotic and, if so, which one. More specific diagnostic information is usually limited to the results of a blood culture (not routinely available in most African hospitals and not available for the critical first 24–48 hours of admission).

Biomarkers to discriminate between blood stream bacterial compared to viral infection are still of limited clinical use. The reasons are that current tests that are available and affordable to most African hospitals lack sufficient sensitivity and specificity for routine use [42]. In non-severe illness, the proportion of children with a blood stream bacterial infection is approximately 1–3% [4,59] so that unless the specificity of a test is high, the predictive value of a positive test will be small and large numbers of antibiotic treatments will be given needlessly. In addition, a large number of tests with significant costs per test will be used to detect a small number of positives. By contrast, in children with severe illness the probability of a bacterial blood stream infection is relatively high and these infections are associated with significant mortality [13,68]. Thus, unless the sensitivity of the RDT is very high, a clinician would not be justified in withholding an antibiotic on the basis of a negative RDT for bacterial infection. The current WHO criteria for antibiotic treatment in severely ill children also suffer from lack of sensitivity and many clinicians would feel justified in prescribing antibiotics to all severely ill children, at least for the first 24–48 hours of admission, until blood culture results are known and during the period in which the majority of deaths in paediatric wards in Africa occur [13,68,69]. There is clearly a need to improve guidelines for care, particularly through incorporating quality-controlled laboratory results or “point of care” diagnostics into routine care.

### 4.2 Disease surveillance and control

There are additional public health reasons for a much more comprehensive knowledge of what infections are prevalent and where. These include the need to guide the deployment of vaccines or other preventive strategies. In addition, sentinel sites can identify emerging infections and, in particular, epidemic or pandemic threats to sub-Saharan Africa. This is increasingly important with growing global travel both into and out of the African continent.

### 4.3 Future trends

Child mortality is declining in Africa due to a number of reasons but the decline in malaria transmission intensity and the introduction of vaccines against Hib and pneumococcal disease are important contributors. There is an additional benefit to malaria control in that up to half of all blood stream bacterial infections in African children are caused by NTS and these infections become rare in children at low levels of malaria transmission, although may still be seen in HIV-infected adults [22,63]. This “added value” to malaria control could well account for the fact that malaria control has often been followed by greater than expected reductions in child mortality [70]. New vaccines, particularly against meningococcal disease and RSV are likely to have additional benefits to child survival in Africa.

These trends are very positive but one worrying effect of improvement in the diagnosis of malaria has been an increase in the prescription of antibiotics, which is likely to increase the already high levels of resistance to affordable antibiotics in Africa [13]. There is a need to more accurately define indications for antibiotic treatment in African children with non-severe illness and to review the use of amoxicillin in children with WHO criteria of “non-severe pneumonia”, especially in a scenario where the incidence of bacterial pneumonia is declining.
